# Gamma irradiation preserves immunosuppressive potential and inhibits clonogenic capacity of human bone marrow-derived mesenchymal stromal cells

**DOI:** 10.1111/jcmm.12264

**Published:** 2014-03-21

**Authors:** Ana Valéria Gouveia de Andrade, Julia Riewaldt, Rebekka Wehner, Marc Schmitz, Marcus Odendahl, Martin Bornhäuser, Torsten Tonn

**Affiliations:** a Institute for Transfusion Medicine, German Red Cross Blood Donation Service North-EastDresden, Germany; b Institute of Immunology, Medical Faculty, Technische Universität DresdenDresden, Germany; c Center for Regenerative Therapies DresdenDresden, Germany; d Department of Medicine I, University HospitalDresden, Germany; e Transfusion Medicine, Medical Faculty, Technische Universität DresdenDresden, Germany

**Keywords:** mesenchymal stromal cells, gamma irradiation, immunosuppression, GvHD

## Abstract

Mesenchymal stromal cells (MSCs) are promising candidates for the treatment of graft-versus-host and autoimmune diseases. Here, by virtue of their immunosuppressive effects, they are discussed to exhibit inhibitory actions on various immune effector cells, including T lymphocytes that promote the underlying pathology. While it becomes apparent that MSCs exhibit their therapeutic effect in a transient manner, they are usually transplanted from third party donors into heavily immunocompromised patients. However, little is known about potential late complications of persisting third party MSCs in these patients. We therefore analysed the effect of gamma irradiation on the potency and proliferation of MSCs to elucidate an irradiation dose, which would allow inhibition of MSC proliferation while at the same time preserving their immunosuppressive function. Bone marrow-derived MSCs (BM-MSCs) were gamma-irradiated at increasing doses of 5, 10 and 30 Gy and subsequently assessed by colony formation unit (CFU)-assay, Annexin V-staining and in a mixed lymphocyte reaction, to assess colony growth, apoptosis and the immunosuppressive capacity, respectively. Complete loss of proliferative capacity measured by colony formation was observed after irradiation with a dose equal to or greater than 10 Gy. No significant decrease of viable cells was detected, as compared to non-irradiated BM-MSCs. Notably, irradiated BM-MSCs remained highly immunosuppressive *in vitro* for at least 5 days after irradiation. Gamma irradiation does not impair the immunosuppressive capacity of BM-MSCs *in vitro* and thus might increase the safety of MSC-based cell products in clinical applications.

## Introduction

Mesenchymal stromal cells (MSCs) are mesoderm-derived multipotent cells that are increasingly used in novel therapeutic strategies because of their intrinsic immunomodulatory, anti-inflammatory and regenerative properties [1–3]. Especially, their immunosuppressive capacities render MSC-based therapeutics an attractive option for the prevention and treatment of graft-versus-host-disease (GvHD) and the treatment of autoimmune diseases [4,5].

Graft-versus-host-disease is one of the major complications following allogeneic hematopoietic stem cell transplantation (aHSCT) [6], and a major cause of non-relapse morbidity and mortality which affects up to 60% of aHSCT patients and accounts for approximately 15% of deaths after aHSCT [7–9]. The main, if not exclusive inducers of GvHD are donor-derived αβ T cells that recognize the recipient's tissues as ‘non-self’ and attack them by employing a wide range of immune mechanisms [10–12]. Glucocorticoids are the gold standard therapy for acute and chronic GvHD. However, the results of glucocorticoid treatment are clearly suboptimal with regard to efficiency and occurrence of severe adverse effects, with continuing response rates of only 20–40% in both forms of GvHD [13,14].To increase the response rate of the initial treatment and/or to reduce glucocorticoid exposure, studies tested the efficacy of co-administration of other immunosuppressant agents (*i.e*., antithymocyte globulin, mycophenolate mophetil) with glucocorticoids [15]. As alternative treatments, novel drugs or treatment regimens to increase immunosuppression as well as immunomodulatory procedures such as extracorporeal photopheresis may induce remission of GvHD, but the use of these strategies may lead to infectious complications or other adverse effects. Thus, general immunosuppressants currently remain the standard treatment. However, particularly for patients with high-risk features more effective and less toxic therapies are warranted [16].

An alternative approach to the mentioned conventional therapies is the therapeutic use of MSCs, which has been shown in a broad spectrum of recent studies. Notwithstanding, clinical trials yielded ambiguous results on the effects of MSCs. A study conducted in the United States with *in vitro* expanded BM-MSCs (Prochymal, the world's first FDA approved stem cell therapy, Osiris Therapeutics) failed to show any effectiveness in two phase III clinical trials for GvHD [17]. On the other hand, European studies using third party MSCs obtained significant response rates and improved outcome in the prevention and/or treatment of acute and chronic GvHD [18–24]. These rather ambiguous findings might result from insufficient standardization during the MSC isolation, expansion and administration procedures and interindividual MSC donor differences.

Of note, MSCs have been discussed to harbour the risk of ectopic tissue formation [25,26]. Kramann *et al*. transplanted MSCs in rat models of chronic kidney disease and showed that MSCs undergo osteogenic differentiation [25], whereas Breitbach *et al*. observed calcification/ossification areas in infarcted hearts of mice injected with MSCs [26]. Furthermore, the issue of a potential malignant transformation of MSC grafts and suitable quality control parameter is currently heavily discussed among clinicians and regulatory authorities [27–29]. Although the risk of malignant transformation seems not to be as high as initially anticipated, rigorous quality controls for genomic stability such as karyotyping on each single batch of MSCs have been suggested to further minimize the risk [27–29]. Nevertheless, protocols suitable to mitigate the risk for ectopic calcification and secondary tumours that might occur as a result of long-term engraftment and differentiation or malignant transformation of third party MSCs are warranted.

It is well-established that irradiation of cellular blood products by using a gamma ray source inactivates T cells [30–33] and inhibits their engraftment, thus decreasing the risk of GvHD development in the transfused patient. In fact, a study carefully titrating gamma irradiation on red blood cell units showed a reciprocal log reduction in T cell proliferation capacity upon increasing ray doses, with undetectable T-cell proliferation at doses equal to or greater than 25 Gy [15]. In addition, recent experiments clearly documented the inhibitory effect of ionizing radiation also on proliferation of MSCs [34,35]. However, the implication of this treatment on their immunosuppressive potential has so far been not assessed. As this appears a particularly relevant question with regard to clinical applications, we here report on experiments designed to address this issue, with the ultimate goal to improve the biosafety of functional MSCs. Thus, we aim to facilitate their clinical utilization in many indications where the potency of MSCs may rely on a bystander effect rather than engraftment of the transplanted cells.

## Material and methods

### Bone marrow and peripheral blood collection

Bone marrow samples were collected from healthy donors (age 21–51 years) at the University Hospital Dresden, after obtaining informed consent. Peripheral blood samples (*n* = 10) were collected from healthy donors at the German Red Cross Blood Donation Service North-East, Dresden, after obtaining informed consent. This study was approved by the local institutional review board.

### Isolation and expansion of BM-MSCs

Bone marrow aspirates were diluted with PBS (Biochrom, Berlin, Germany). Aliquots were layered over Biocoll separating solution (*d* = 1073 g/ml; Biochrom) and centrifuged (980 × g, 20 min., room temperature, slow acceleration/no braking). Mononuclear cells at the interface were recovered and washed twice with PBS. Cells were seeded at a concentration of 6 × 10^5^ cells/cm^2^ in T175 flasks (Greiner Bio-One, Frickenhausen, Germany) containing DMEM (Life Technologies, Darmstadt, Germany) supplemented with GlutaMAX-I™ 2 mM (Life Technologies) and 10% foetal bovine serum (FBS; Biochrom) and grown at 37°C in 5% CO_2_. Non-adherent cells were removed after 24 hrs by washing with PBS. Medium was changed every 2–3 days until cells reached 80% confluency and were harvested by using Trypsin-EDTA solution (0.5 g/l porcine trypsin with 0.2 g/l EDTA·4Na; Sigma-Aldrich; Schnelldorf, Germany). All BM-MSCs were characterized according to the criteria defined by the International Society of Cellular Therapy (ISCT; see below ‘Immunophenotypical characterization’ and ‘Differentiation potential’) [36]. For flow cytometric and functional analysis, MSCs from the second to fourth passage were used.

### Isolation of peripheral blood mononuclear cells (PBMCs)

Peripheral blood samples were diluted 1:2 with PBS. Diluted samples were layered over Biocoll separating solution and centrifuged as described above. Mononuclear cells at the interface were recovered and washed twice with PBS. Harvested cells were resuspended in RPMI 1640 medium (Biochrom) supplemented with 2 mM l-glutamine, 1 mM sodium pyruvate, 1% nonessential amino acids, 100 μg/ml penicillin, 100 μg/ml streptomycin and 10% FBS (all from Biochrom) and counted. Aliquots were stored at −80°C in freezing medium (supplemented RPMI 1640 + 10% DMSO, Wak-Chemie Medical GmbH; Steinbach/Ts, Germany).

### Immunophenotypical characterization of MSCs

To assess the immunophenotypical profile of BM-MSCs, monoclonal antibodies (clones) to the following surface antigens were used: CD73 (AD2), CD90 (5E10), CD105 (43A4E1), CD45 (HI30), CD31 (WM59), HLA-DR (G46-6), HLA-ABC (DX17), CD39 (eBioA1), conjugated to APC, PE, FITC, Biotin, V450, V500 and PE-Cy7, as well as APC-Cy7-conjugated Streptavidin were purchased from BD Biosciences (Heidelberg, Germany), Miltenyi Biotec (Bergisch Gladbach, Germany) or eBioscience (Frankfurt, Germany). 10^5^ cells were incubated with fluorochrome-conjugated antibodies for 15 min. at 4°C. BM-MSCs were washed twice, centrifuged for 10 min. at 200 × g at 4°C, resuspended in 300 μl of PBS and analysed by using a FACS Canto II (BD Biosciences). Data were analysed by using the FlowJo software (TreeStar Inc., Ashland, OR, USA).

### Differentiation potential of BM-MSCs

Subconfluent (80% of confluency) MSCs of different lines (*n* = 4) were seeded at 2 × 10^4^ MSCs/cm^2^ into 24-well plates with 1 ml of DMEM + 10% FBS. After 24 hrs, DMEM-medium was replaced by STEMPro Osteogenesis Differentiation medium and STEMPro Adipogenesis Differentiation medium (both from Life Technologies) for differentiation into osteoblasts and adipocytes, respectively. Cells were cultured at 37°C and 5% CO_2_ with media exchange every 2–3 days. Osteoblastic differentiation of MSCs was determined by Alizarin Red (Sigma-Aldrich) staining after 22 days, and adipocytic differentiation by Oil Red O (Sigma-Aldrich) staining after 18 days of culture.

### Irradiation of BM-MSCs

BM-MSCs were irradiated in suspension in tubes by using the Gammacell 3000 Elan device (Best Theratronics, Ottawa, ON, Canada) and doses of 5, 10 or 30 Gy. In the meantime, non-irradiated controls were kept at room temperature. Both irradiated and non-irradiated MSCs were washed and subjected to further assays. For the immunosuppressive assay performed over time, cells were irradiated within the flasks, washed and maintained in culture until the harvesting day.

### Colony forming unit (CFU) assay

BM-MSCs (*n* = 2) were harvested upon reaching 80–90% confluency, irradiated as described above, washed, counted and plated (50 BM-MSCs/cm^2^ in 6-well plates, *i.e*. 500 cells/well) with 2 ml of StemMACS MSC Expansion Medium human (Miltenyi Biotec). Plates were incubated at 37°C and 5% CO_2_ for 14 days for colony formation. After that, MSCs were stained with Crystal Violet 0.5% (Sigma-Aldrich) for 5 min. at room temperature and washed. For better colony visualization, 2 ml of PBS were added to each well. Pictures were taken with the STEMvision equipment (Stem Cell Technologies, Vancouver, BC, Canada) and colonies were counted. For a larger-scale assay, different BM-MSC lines (*n* = 3) were plated at 60 cells/cm^2^ (*i.e*. 10^4^ cells/flask) in T175 flasks, incubated for 4 weeks and stained with Crystal Violet 0.5% as described above. Because of colony overgrowth, non-irradiated control cultures were already stained on day 16.

### Mixed Lymphocyte Reaction (MLR)

To test MSCs for their *in vitro* immunosuppressive capacity, MLRs were performed. For that, a stimulator and a responder cell stock were generated by isolating PBMCs with a density gradient from 9 (pooled) and 1, respectively, donor blood samples as described above. For the MLR, each 5 × 10^3^ BM-MSCs from different lines (*n* = 3) were plated in 96-well round bottom plates (TPP, Biochrom). Incubation at 37°C, 5% CO_2_ for 1 hr allowed MSCs adherence. Afterwards, stimulator cells were thawed, irradiated with 30 Gy, washed and plated at 10^5^ cells/well. Thawed responder cells were added at 1:1 ratio to the stimulator cells in a final volume of 200 μl supplemented RPMI 1640 medium. Cultures were incubated for 5 days at 37°C and 5% CO_2_. 1 μCi ^3^H-thymidine (Hartmann Analytic, Braunschweig, Germany) was added to the culture on day 5. After 18 hrs, cells were harvested by using the Inotech Cell Harvester (Inotech Biosystems International Inc., Derwood, MD, USA). ^3^H-thymidine incorporation was measured with the 1450 MicroBeta TriLux scintillation counter (Perkin Elmer Life Sciences, Rodgau-Jügesheim, Germany), giving the level of radioactivity as ‘Corrected Counts per Minute’. For evaluation of immunosuppressive capacity over time, BM-MSCs were plated at the same day and irradiated at different time-points (24, 48, 72, 96 and 120 hrs). The harvesting of all irradiated BM-MSCs and the non-irradiated control cells was performed 5 days after plating.

### Apoptosis assay

Cell apoptosis was assessed with the FITC Annexin V Apoptosis Detection Kit I (BD Biosciences) according to the manufacturer's instructions. Briefly, BM-MSC lines (*n* = 3) were harvested, washed and resuspended in binding buffer. After adding Annexin V-FITC and propidium iodide (PI), cells were incubated for 15 min. at room temperature. Data were acquired with a FACSCanto II (BD Biosciences) and analysed by using the FlowJo software (Treestar Inc.).

### Statistical analysis

Statistical significance was assessed by using the GraphPad Prism software (version 5.04; Graphpad, San Diego, CA, USA) and the unpaired Student's *t*-test. Differences were considered significant when *P* < 0.05, with *P < 0.05, **P < 0.01, ***P < 0.001 and ****P < 0.0001.

## Results

### Characterization of isolated BM-MSCs

**Figure 1:**
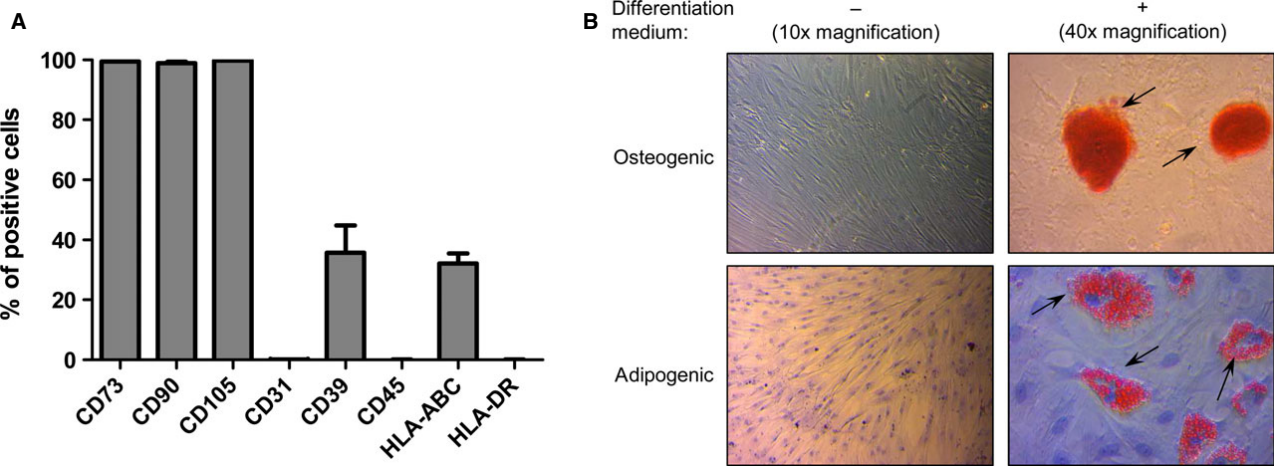
Immunophenotypical profile and differentiation capacity of BM-MSCs. BM-MSCs from different cell lines (*n* = 4, passage 4–6) were characterized. (A) Different surface markers (CD73, CD90, CD105, CD45, CD31, HLA-DR, HLA-ABC and CD39) were analysed by flow cytometry. Bars represent mean percentage of positive cells ±SEM. (B) Differentiation potential into osteoblasts and adipocytes was assessed by the presence of red-stained calcium precipitates and lipid droplets (indicated by arrows; 40× magnification), as visualized by Alizarin Red and Oil Red O staining, respectively. Non-differentiated control cultures are shown in 10× magnification.

We initially sought to confirm the identity of BM-MSCs by flow cytometry. As expected, and consistent with criteria defined by the ISCT [36], all cell lines tested (*n* = 4) exhibited a immunophenotypic profile typical for MSCs. This was reflected in expression of the main MSC surface markers CD73, CD90 and CD105 (mean proportion of cells positive for the respective markers ≥98.9%; Fig.1A). In addition, the isolated cell lines expressed HLA-ABC (mean proportion of positive cells/cell line = 32.2 ± 3.3%; Fig.1A), whereas expression of HLA-DR, CD45 and CD31 was virtually absent (mean proportion of positive cells for each of the respective markers <0.1%; Fig.1A). Furthermore, all cell lines tested were positive for CD39 (35.7 ± 9.1% positive cells; Fig.1A). Together, these data clearly demonstrated that the isolated cells were indeed MSCs.

Next, we assessed the ability of BM-MSCs to differentiate into osteoblasts and adipocytes, a hallmark of this cell type [36], by culturing them in osteogenic and adipogenic differentiation-inducing medium. At day 21 after initiation of culture, osteogenic induction resulted in occurrence of Alizarin Red-positive precipitants in all tested BM-MSC lines (*n* = 4), indicating the presence of osteoblasts (Fig.1B). Similarly, adipogenic induction resulted in the presence of Oil Red O-positive cells at day 14 of culture, indicating successful differentiation into adipocytes (Fig.1B). In non-induced control cultures no osteoblast or adipocyte development was detected, as indicated by the absence of Alizarin Red- and Oil Red O-positive cells (Fig.1B). These results confirmed the ability of BM-MSCs to differentiate into osteoblasts and adipocytes, and absence of spontaneous osteo- or adipogenic differentiation under non-inducing conditions.

### Irradiation dose of 10 Gy is sufficient to inhibit colony formation ability of BM-MSCs

**Figure 2:**
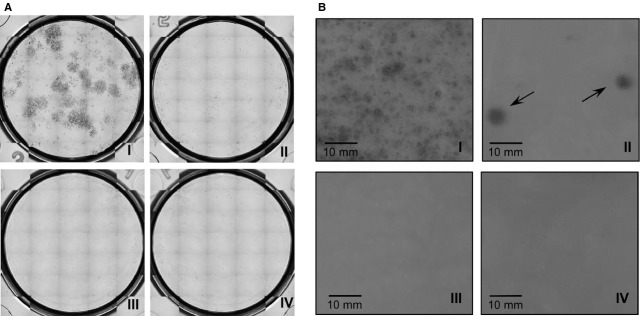
Colony-forming ability of BM-MSCs after gamma irradiation. BM-MSCs from different cell lines were either left untreated or submitted to 5 (II), 10 (III) or 30 Gy (IV) of gamma irradiation and subjected to a CFU assay. Colony formation was assessed by staining with Crystal Violet. (A) Small-scale CFU assay by using 500 BM-MSCs (*n* = 2, passage 4)/well in triplicates and cultivation for 14 days. (A) Representative pictures of cultures. (B) Large-scale CFU assay by using BM-MSCs from different cell lines (*n* = 3, passage 2) by using T175 culture flasks and cultivation for 4 weeks. Pictures show representative detail of cultures, with non-irradiated cultures being stained with Crystal Violet already after 16 days.

Ionizing radiation was previously shown to successfully inhibit proliferation of lymphocytes [37] and reduce MSC proliferation [34,35], as assessed for the latter by conventional cell counting. However, to our knowledge the irradiation sensitivity of MSCs with regard to their potential to form colonies has so far not been shown. Thus, we established a small-scale CFU assay by using different isolated BM-MSCs lines (*n* = 2, passage 4) that were irradiated with 5, 10 or 30 Gy. After cultivation for 2 weeks, non-irradiated BM-MSCs yielded 33.7 ± 4.7 colonies/well (Fig.2A, I). In contrast, a dose equal to or greater than 5 Gy was sufficient to completely abrogate CFU formation (Fig.2A, II–IV). The use of high, although strongly varying doses (0.4–9 × 10^6^/kg bodyweight) of MSCs in clinical settings [18,20,38] increases the probability to observe rare cell clones that retain their proliferative capacity after irradiation, as compared to the small-scale CFU assay. Thus, we assessed colony formation abilities within a higher number of cells by using a large-scale setting and prolonged cultivation (4 weeks) to allow outgrowth of individual irradiation resistant clones. Culture flasks containing non-irradiated, *i.e*. control BM-MSCs exhibited massive colony overgrowth already after 16 days (Fig.2B, I). Consistent with the results from the smaller-scale setting (Fig.2A, III, IV), irradiation with 10 and 30 Gy led to complete abrogation of colony formation (Fig.2B, III, IV). However, irradiation of BM-MSCs with 5 Gy – a dose that was sufficient to completely abrogate colony formation in the small-scale assay – led to formation of 6.7 ± 2.4 CFU/flask (Fig.2B, II), demonstrating the importance of large-scale assays in detecting rare colonies (summarized in Table1).

**Table 1:** MSC colony forming potential

Colony assay	Gamma radiation doses (Gy)			
	0	5	10	30
Small-scale	++++	–	–	–
Large-scale	+++++	+	–	–

Number of colonies is given as: –, no colonies; +, <10 colonies; ++, <20 colonies; +++, <30 colonies; ++++, <40 colonies; +++++, >50 colonies.

### Induction of apoptosis in irradiated BM-MSCs

**Figure 3:**
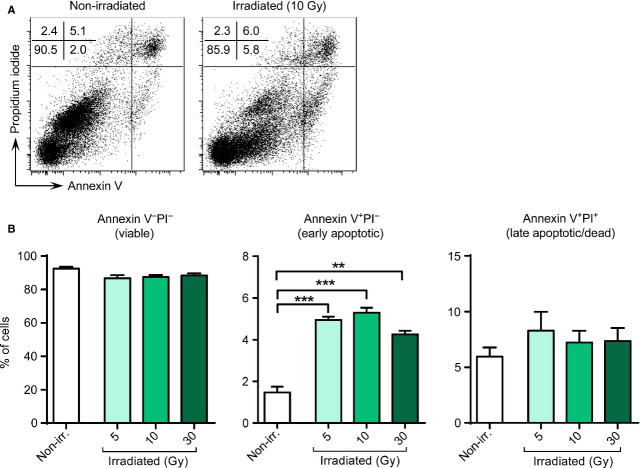
Induction of programmed cell death in gamma-irradiated BM-MSCs. BM-MSCs (*n* = 3, passage 2) were either left untreated or submitted to different doses of gamma irradiation (5, 10 and 30 Gy) and subsequently maintained in culture. After 3 weeks, cells were harvested and stained with Annexin V and propidium iodide (PI). (A) FACS plots from a representative non-treated (left) and 10 Gy-irradiated (right) BM-MSC line. (B) Graphs depicting proportion of viable, non-apoptotic (Annexin V^−^PI^−^), early apoptotic (Annexin V^+^PI^−^), and late apoptotic or dead (Annexin V^+^PI^+^) cells. Data are representative of three independent experiments. Bars represent mean ± SEM (Student's *t*-test: ***P < 0.001, **P < 0.01).

To evaluate the degree of cell damage that resulted from irradiation, we performed an apoptosis assay. To that end, irradiated BM-MSCs and non-irradiated control samples were kept in culture for 3 weeks and subsequently subjected to a staining with Annexin V and PI (Fig.3A). The vast majority of both non-irradiated and irradiated BM-MSCs were Annexin V^−^PI^−^ (mean frequencies: non-irradiated, 92.6 ± 1.1%; 5 Gy, 86.7 ± 1.9%; 10 Gy, 87.5 ± 1.2%; 30 Gy, 88.4 ± 1.2%; Fig.3B, left), indicating a viable and non-apoptotic state. In contrast, gamma irradiation led to a slight increase in Annexin V^+^PI^−^ cell frequencies (non-irradiated, 1.5 ± 0.3%; 5 Gy, 4.9 ± 0.2%; 10 Gy, 5.3 ± 0.2%; 30 Gy, 4.3 ± 0.2%), suggesting a dose-independent, mild induction of early apoptosis in BM-MSCs, as compared to non-irradiated samples (Fig.3B, middle). However, frequencies of Annexin V^+^PI^+^, *i.e*. late apoptotic or dead, cells remained stable irrespective of whether cells had been subjected to radiation or not (Fig.3B, right). In summary, these data demonstrate that gamma irradiation does not significantly reduce the numbers of viable cells.

### Preserved immunosuppressive capacity of irradiated BM-MSCs

**Figure 4:**
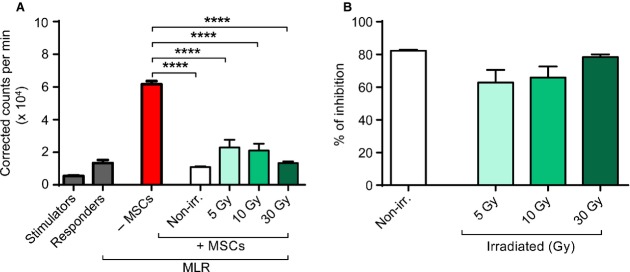
Immunosuppressive properties of gamma-irradiated BM-MSCs. Responder and stimulator cells were co-cultured (MLR) in the absence or presence of BM-MSCs (passage 2, *n* = 3) that were left untreated or submitted to different doses of gamma irradiation (5, 10 and 30 Gy). Proliferation was assessed at day 6 of culture by measurement of ^3^H-thymidine incorporation. (A) Graph depicts ^3^H-thymidine incorporation into stimulator and responder cells alone, together and in co-culture with non-irradiated or irradiated BM-MSCs, as indicated. Experiments were performed in triplicates. (B) Graph depicts proportional inhibition of lymphocyte proliferation within the MLR. Bars represent mean ± SEM. Non-irr., non-irradiated. (Student's *t*-test: ns, *P* > 0.05; ****P < 0.0001).

In the clinical context, the MSC function of main interest is their immunomodulatory potential. As it is therefore crucial to evaluate the immunosuppressive capacity of irradiated BM-MSCs, we established a standardized MLR assay. Here, co-cultivation of stimulator and responder cells, *i.e*. a MLR, yielded strong and reliable proliferation (Fig.4A). Upon addition of non-irradiated BM-MSCs to the MLR, the lymphocyte proliferation was significantly reduced by >5-fold, clearly demonstrating MSC-mediated inhibition of proliferation. Of note, despite incapacitation of BM-MSC proliferation (Fig.2) and mild increase in early apoptotic cell frequencies (Fig.3) by irradiation with increasing doses of gamma rays, the inhibitory effect mediated by BM-MSCs on lymphocyte proliferation remained highly significant, as compared to the cultures that did not contain MSCs (Fig.4A). Inhibition of lymphocyte proliferation in MLRs mediated by irradiated BM-MSCs (5 Gy: 62.8 ± 7.7%, 10 Gy: 65.8 ± 6.8%, 30 Gy: 78.4 ± 1.7%) was similar to that observed in MLRs containing non-irradiated BM-MSCs (82.2 ± 0.7%; Fig.4B).

**Figure 5:**
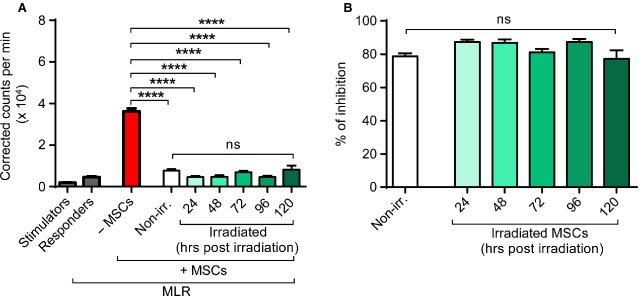
Sustained immunosuppressive capacity of BM-MSCs 5 days after irradiation. BM-MSCs (passage 4, *n* = 3) were submitted to a dose of 10 Gy of gamma irradiation and maintained in culture. A MLR in triplicates was set up in the absence or presence of BM-MSCs that were either left untreated or were irradiated with 10 Gy 24, 48, 72, 96 or 120 hrs ago, as indicated. Proliferation was assessed by measurement of ^3^H-thymidine incorporation. (A) Graph depicts thymidine incorporation of stimulator and responder cells alone, together and in co-culture with non-irradiated BM-MSCs or BM-MSCs at varying time-points after irradiation, as indicated. (B) Graph depicts proportional inhibition of lymphocyte proliferation within the MLR. Bars represent mean ± SEM (Student's *t*-test: ****P < 0.0001).

To address the question whether the preservation of immunosuppressive properties might only be a temporary effect, we performed time course experiments. Assessing the impact of BM-MSCs (*n* = 3) on a MLR at 24, 48, 72, 96 and 120 hrs after their irradiation with 10 Gy, we could demonstrate that their immunosuppressive potential was comparable to non-irradiated BM-MSCs over time (Fig.5A). Similar to non-irradiated controls (mean inhibition 78.7 ± 1.85%), irradiated BM-MSCs yielded a mean inhibition of 77.3 ± 5.1% after 5 days (Fig.5B). Thus, our results showed sustained and stable immunosuppressive capacities of gamma-irradiated BM-MSCs at a dose of 10 Gy for at least 5 days after irradiation *in vitro*.

## Discussion

In view of patient safety, clinical applications of MSCs, even in compassionate use situations, should consider the potential risks such as secondary engraftment, which might lead to ectopic tissue formation [25,26] or tumour formation caused by malignant transformation [5]. So far, and despite observations of osteogenic differentiation in kidneys and calcification/ossification areas in hearts of MSC-transplanted rodents [25,26], ectopic tissue formation after MSC therapy has not been shown in humans. Likewise, up to date no MSC-originating tumours have been diagnosed in patients who underwent MSC therapy [28] and malignant transformation of *in vitro* expanded human MSCs is estimated to be a rather uncommon event (frequency <10^−9^) [27]. Nevertheless, there are contradictory data published so far on the potential engraftment and long-term persistence of third party MSCs and thus on potential late risks attributed to donor chimerism in recipients. These microchimerism of long-term persisting allogeneic cell population, *e.g*. after transfusion of blood products, might be involved in long-term complications including the development of autoimmune-like symptoms or chronic GvHD [39,40]. Yet, PCR analysis of various tissue autopsies from MSC recipients showed very low or no donor chimerism [41], indicating a rejection of allogeneic MSCs by the recipient's immune system. However, by using sensitive qRT-PCR techniques, some studies were able to detect MSC donor chimerism in various tissues (*i.e*. bone marrow, bladder, lymph nodes and intestine) of the recipient [41–43], even 120 days after MSC infusion [42]. Thus, one should take into account that the detection of low-level donor chimerism is difficult to be achieved and it seems to be of great importance to establish detection methods with higher sensitivity to assess low-level MSC chimerism in transplanted patients. It may be very likely that MSCs are prone to long-term persistence in the recipient because of their ability to escape an immune surveillance in the host. In addition, to date MSCs are often transfused into immunocompromised patients (*e.g*. after an allogeneic stem cell transplantation), whose immune system is less equipped to prevent a potential MSC engraftment. Hence, immunosuppressed patients are also more prone to tumour development [44].

It is therefore critical to minimize the engraftment potential of transplanted MSCs. Furthermore, reliable quality control parameters for malignant transformation of MSCs and the use of processing methods that avoid the risk of karyotypic changes (slow MSC growth and short expansion times 28) are warranted. Interestingly, it is suggested that the described modulatory capacities on immune responses and pro-regenerative effects of MSCs are mediated by a ‘hit-and-run’ bystander mechanism, rather than by long-term engraftment of MSCs at the site of injury [41]. However, a recent report by Meleshko *et al*. [42], describing MSC donor chimerism even 120 days after MSC infusion, implicated the need for an approach to minimize the engraftment ability of BM-MSCs (*e.g*. by inhibition of the closely linked proliferation potential), with the requirement to maintain the immunomodulatory effect of the cells. In transfusion medicine, good experience with gamma irradiation has been made with respect to inhibition of the proliferative capacity of lymphocytes present within blood products to avoid transfusion-associated GvHD [37]. Here, a mean dose of 30 Gy is recommended [37,45].

For detection of cells that remain viable after irradiation, we performed both short- and long-term CFU assays. In our small-scale CFU assay, we could not detect any colonies after 2 weeks of cultivation of BM-MSCs irradiated with 5 Gy. Although we have obtained comparable results for various different donors, we cannot exclude that interindividual differences with regard to the sensitivity may influence the potency. Especially in view of patient-derived MSCs, who may have experienced extensive oncologic pre-treatment, the optimal irradiation dose has to be validated. In contrast, longer cultivation (4 weeks) of higher BM-MSC numbers that were irradiated with 5 Gy (up-scaled assay) allowed sporadic growth of colonies. From these data, we concluded that higher numbers of to-be-tested cells as well as longer cultivation periods increase the sensitivity of this assay to detect remaining viable and proliferating MSCs after irradiation. Taking into consideration that for cell therapy, high doses (0.4–9 × 10^6^/kg bodyweight) of MSCs are being employed [18,20,38], knowledge obtained from large-scale experiments about the frequencies of remaining proliferative MSCs is of great importance. From our experience, we recommend to use a dosage of 10 Gy to inhibit proliferation and decrease engraftment potential of MSCs and thus to increase therapeutic safety. However, our data suggest that, if necessary, a further increased irradiation dose (*e.g*. of 30 Gy) might be applied without negative impact on the immunosuppressive capacity of MSCs.

Upon irradiation, various types of stem cells (*e.g*. embryonic stem cells, hematopoietic stem cells and intestinal stem cells) undergo extensive apoptosis to eliminate cells that acquired harmful genetic defects [46–48], thereby reducing the risk of both developmental problems and carcinogenesis. Other stem cell types, such as keratinocyte stem cells and bulge stem cells, are relatively resistant to irradiation induced cell death [49,50]. Confirming previous reports on mouse and human MSCs [35,51], we observed in all dosages tested only minimal apoptosis in BM-MSCs following radiation exposure. Consistently, Cmielova *et al*. [52] demonstrated that although gamma irradiation leads to stress-induced premature senescence in MSCs isolated from bone marrow, periodontal ligaments and dental pulp, senescent MSCs remain viable. Importantly, absence of massive apoptosis induction in irradiated MSCs is a critical parameter with regard to clinical application. According to our data, irradiation of MSCs with dosages in the range of 10–30 Gy would not result in administration of significantly increased apoptotic cell numbers into the patients.

According to previously published data, ionizing radiation affects functional properties of MSCs. Li *et al*. [34] showed that X-ray radiation exerts a sustained inhibitory effect on MSC growth and osteogenic differentiation potential for up to 2 weeks post-exposure, while the adipogenic differentiation potential seemed to be more resistant to irradiation. Noteworthy, and to our knowledge for the first time, our results clearly demonstrated sustained immunosuppressive capacities of human irradiated BM-MSCs. After gamma irradiation with a dose of 10 Gy, their immunosuppressive potential remained on a high level for 5 days. The sustained immunomodulatory activity is highly relevant for the use of MSCs in the clinical context, where time-consuming quality tests on the to-be-transferred cell population might be required. Importantly, a potential clinical application of gamma-irradiated MSCs requires the adaptation of the culturing protocols to GMP-compliant conditions, *e.g*. the use of certified FBS batches or alternatively platelet lysate, and animal origin-free trypsin-like enzymes instead of FBS and trypsin, respectively. Possible mechanisms that may facilitate MSC radio-resistance are ATM protein phosphorylation, activation of cell-cycle checkpoints, double-strand break repair by homologous recombination and non-homologous end joining, and antioxidant capacity to scavenge reactive oxygen species [35]. Of note, when compared to a cancer cell line shown to be radio-resistant, MSCs exhibited similar survival curves and cell-cycle changes as well as a high capacity to scavenge reactive oxygen species by antioxidants and active double-strand break repair [35]. In addition, susceptibility of cells to radiation-induced damages might depend on their anatomical origin. Studies evaluating bone marrow stromal cells from maxilla, mandible and iliac crest showed better recovery of orofacial MSCs after therapeutically relevant radiation doses (0–10 Gy), as compared to that of MSC from the iliac crest [53].

With the here presented experiments, we demonstrated that gamma irradiation with a single dose of 10 Gy is sufficient to inhibit BM-MSC proliferation, whereas their immunomodulatory potential is preserved for at least 5 days thereafter. Based on this, we believe that pre-transfer irradiation of MSC-based cell products might be a reasonable approach to increase their safety in clinical applications, *e.g*. in the treatment of GvHD or autoimmune disorders.
